# New *Metalimnobia* crane flies (Diptera, Limoniidae) from China with an update of species distributions

**DOI:** 10.3897/zookeys.1008.60704

**Published:** 2020-12-31

**Authors:** Ye Jiang, Xiao Zhang

**Affiliations:** 1 Key Lab of Integrated Crop Pest Management of Shandong Province, College of Plant Health and Medicine, Qingdao Agricultural University, Qingdao 266109, China Qingdao Agricultural University Qingdao China

**Keywords:** Classification, distribution, identification key, Limoniinae, Limoniini, new species, taxonomy

## Abstract

Two new species of the genus *Metalimnobia* Matsumura, 1911 from southwest China, M. (M.) bicolor**sp. nov.** and M. (M.) caudifusca**sp. nov.**, are described and illustrated. The new species can be distinguished from congeners by their wing patterns and male genitalia. New distributional data are given for the following species: M. (M.) bifasciata (Schrank, 1781), M. (M.) quadrinotata (Meigen, 1818) and M. (M.) tenua Savchenko, 1976. An updated key for all Chinese *Metalimnobia* crane flies is presented.

## Introduction

*Metalimnobia* Matsumura, 1911 is a small genus in the family Limoniidae with 48 known species/subspecies. The genus is characterized by the following characters: body medium-sized to large, wing length more than 10 mm; antenna with 12 or 13 flagellomeres, palpus with five segments; wing distinctly patterned with dark brown spots or markings and often with smoky areas; R_1+2_ and R_2_ short and transverse, almost ending at the same level, basal section of CuA_1_ before the fork of M; male gonocoxite with a large ventro-mesal lobe, inner gonostylus often divided into three lobes; ovipositor with a short and small cercus and large hypovalva (Dienske 1987; Podenas and Gelhaus 2007; Salmela and Starý 2009; Mao and Yang 2010; Podenas and Byun 2016).

Members of the genus *Metalimnobia* are grouped into three subgenera: *Metalimnobia* (s. str.) (34 species/subspecies), *Tricholimonia* Alexander, 1965 (11 species) and *Lasiolimonia* Alexander, 1976 (three species). The nominotypical subgenus is known from the Palaearctic (15 species/subspecies), Nearctic (12 species) and Oriental (11 species/subspecies) regions. The other two subgenera are known only from the Afrotropical region (Oosterbroek 2020).

Nine *Metalimnobia* crane flies all belonging to the nominotypical subgenus have been recorded from China (Oosterbroek 2020), of which five were published by Mao and Yang (2010). In this paper, two new species are added to the Chinese fauna. Examination of specimens from several localities in China also revealed new distribution records for M. (M.) bifasciata (Schrank, 1781), M. (M.) quadrinotata (Meigen, 1818) and M. (M.) tenua Savchenko, 1976. A dichotomous key modified from Mao and Yang (2010) for all Chinese *Metalimnobia* crane flies is given.

## Materials and methods

Specimens for this study were collected from several localities in China by different entomologists in the period 2009–2019. Adult crane flies were collected by insect net, Malaise trap and light trap. Type specimens of known *Metalimnobia* species deposited in the National Museum of Natural History, Smithsonian Institution, Washington, DC, USA (**USNM**), the Natural History Museum, London, UK (**NHM**) and the Entomological Museum of China Agricultural University, Beijing, China (**CAU**) were also examined. Type specimens of the new species were deposited in CAU. Other specimens were deposited in the Entomological Museum of Qingdao Agricultural University, Shandong, China.

Genitalic preparations of males were made by macerating the apical portion of the abdomen in cold 10% NaOH for 12–15 hours. Observations and illustrations were made using a ZEISS Stemi 2000-C stereomicroscope. Photographs were taken with a Canon EOS 77D digital camera through a macro lens. The morphological terminology mainly follows McAlpine (1981), and the venation is described after Alexander and Byers (1981).The following abbreviations in figures are used: **tg 10** = tenth tergite, **st 9** = ninth sternite, **goncx** = gonocoxite, **o gonst** = outer gonostylus, **i gonst** = inner gonostylus, **aed** = aedeagus, **pm** = paramere, **cerc** = cercus, **hyp vlv** = hypogynial valve.

## Taxonomy

### Key to Chinese *Metalimnobia* crane flies

**Table:** 

1	Wing yellowish	**2**
–	Wing grayish or brownish (Figs 2, 5d)	**3**
2	Femora yellow with tips brown	**M. (M.) bifasciata (Schrank, 1781)**
–	Femora dark brown with subapical yellow rings	**M. (M.) xanthopteroides xanthopteroides (Riedel, 1917)**
3	Wing with Sc_1_ ending at about 2/3 of Rs, inner gonostylus divided into four lobes	**M. (M.) improvisa (Alexander, 1933)**
–	Wing with Sc_1_ ending close to or beyond fork of Rs (Figs 2, 5d), inner gonostylus divided into three lobes (Figs 3, 6)	**4**
4	Wing without spot at base (Fig. 5d)	**M. (M.) caudifusca sp. nov.**
–	Wing with one or more spots at base (Fig. 2)	**5**
5	Mid and hind femora each with apical ring	**M. (M.) yunnanica (Edwards, 1928)**
–	Mid and hind femora each with two rings (Figs 1a, 5a)	**6**
6	Subapical rings of mid and hind femora as dark and wide as apical rings	**M. (M.) quadrimaculata (Linnaeus, 1760)**
–	Subapical rings of mid and hind femora paler than apical rings and spreading over half of femora (Figs 1a, 5a)	**7**
7	Wing spots at origin and fork of Rs with upper parts brown and lower parts brownish black (Fig. 2)	**M. (M.) bicolor sp. nov.**
–	Wing spots at origin and fork of Rs uniformly dark brown (Fig. 5d)	**8**
8	Pleuron of thorax mostly black, paramere with tuft of hairs at tip (Fig. 4h)	**M. (M.) rectangularis Mao & Yang, 2010**
–	Pleuron of thorax mostly brownish yellow, paramere without hair at tip	**9**
9	Outer gonostylus slender, nearly as wide as innermost lobe of inner gonostylus	**M. (M.) impubis Mao & Yang, 2010**
–	Outer gonostylus broad, about twice as wide as innermost lobe of inner gonostylus	**10**
10	Paramere with tip flattened	**M. (M.) quadrinotata (Meigen, 1818)**
–	Paramere with tip narrow and acute (Fig. 4b, e)	**M. (M.) tenua Savchenko, 1976**

#### Metalimnobia (Metalimnobia) bicolor sp. nov.

Taxon classificationAnimaliaDipteraLimoniidae

ED1D9560-6B2D-517E-8A0B-6B2D58866B73

http://zoobank.org/59181930-7D58-414F-952B-3B4A56E74E0E

Figures 1 , 2 , 3

##### Type material.

***Holotype*** male, China: Sichuan, Batang, Deda (30°17'43"N, 99°23'50"E, 3727 m), 2019.VII.17, Liang Wang. ***Paratypes***: 1 male 1 female, same data as holotype.

##### Diagnosis.

Pleuron brownish black with brownish yellow stripe extending from pronotum to base of wing. Femora each with two rings, apical ring brown, subapical ring slightly paler and spreading over half of femora. Wing brownish with one or more brown spots at base, two large spots at origin and fork of Rs with upper parts brown and lower parts brownish black. Sc_1_ ending beyond fork of Rs, Sc_2_ shorter than Sc_1_; basal section of CuA_1_ before fork of M. Inner gonostylus divided into three lobes. Paramere with distal part long and nearly straight, tip flattened and bare.

##### Description.

**Male. *Body*** length 10.5–10.8 mm, wing length 11.8–12.0 mm.

**Figure 1.**
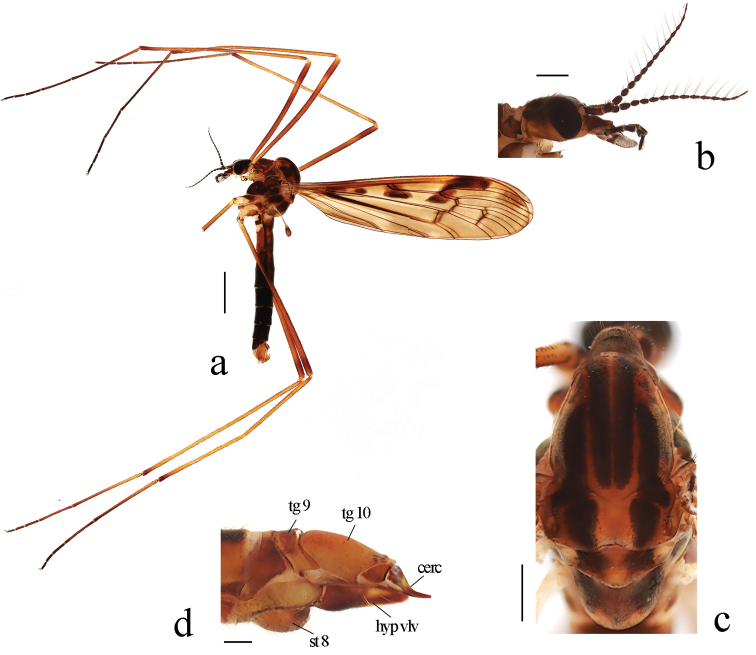
Metalimnobia (Metalimnobia) bicolor sp. nov. **a** male habitus, lateral view **b** head, lateral view **c** thorax, dorsal view **d** ovipositor, lateral view. Scale bars: 2.0 mm (**a**); 0.5 mm (**b, c**); 0.2 mm (**d**).

***Head*** (Fig. 1b) brown with vertex and frons dark brown. Setae on head dark brown. Antenna length 2.5–2.8 mm, brownish black with scape dark brown. Scape cylindrical; pedicel nearly globose; flagellomeres oval with long setae, each flagellomere slightly narrower than previous one, terminal flagellomere elongated. Rostrum dark brown with dark brown setae. Palpus brownish black with brownish black setae.

**Figure 2.**
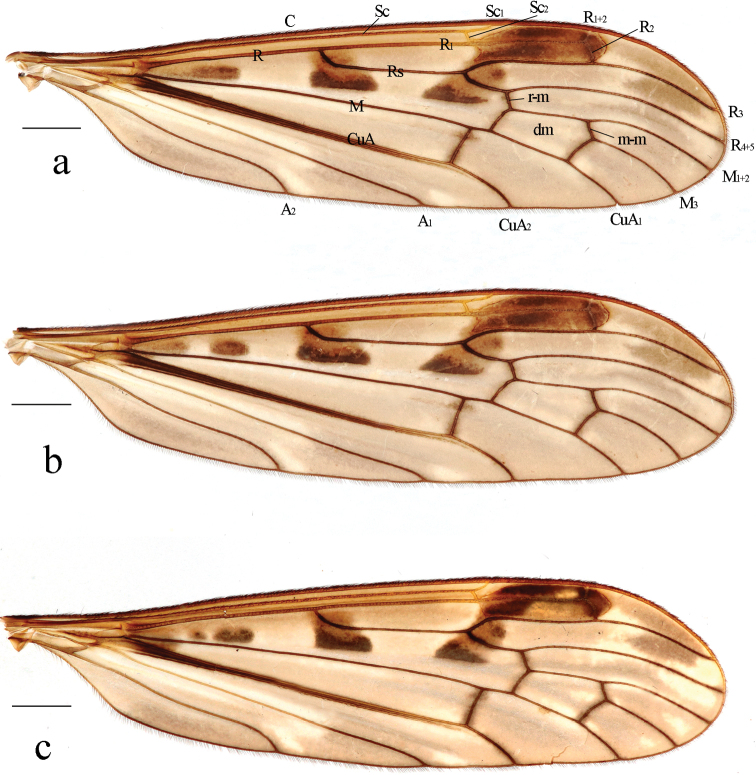
Variations of wing pattern of Metalimnobia (Metalimnobia) bicolor sp. nov. Scale bars: 1.0 mm.

***Thorax*** (Fig. 1c). Pronotum brownish black with lateral margins brown. Prescutum brown with four broad, brownish black longitudinal stripes, two lateral stripes about 1/2 length of median stripes. Scutum brown with middle area brownish yellow; each lobe with two large brownish black spots, anterior spot connected with lateral stripe of prescutum. Scutellum brownish black with middle area brownish yellow. Mediotergite brownish black, posterior area with two nearly confluent pale brownish yellow spots. Pleuron (Fig. 1a) brownish black with a brownish yellow stripe extending from pronotum to base of wing. Setae on thorax brownish black. Coxae brown; trochanters pale yellow; femora each brownish yellow with two rings, apical ring brown, subapical ring slightly paler and spreading over half of femora; tibiae brownish yellow with tip dark brown; tarsi brownish black, basal 1/3–1/2 of first tarsal segments brownish yellow. Setae on legs brownish black. Wing (Fig. 2) brownish with brown to brownish black pattern: long oval, brown spot at wing base (Fig. 2a), often divided into two or three small spots (Fig. 2b, c); two large spots at origin and fork of Rs, each spot with upper part pale brown and lower part brownish black; stigma brown with each side darker; brown seams along cord, m-m, basal section of M_3_ and base of CuA; obscure, irregular brownish clouds in most cells, darker near R_3_. Veins brownish yellow, darker in clouded areas. Venation: Sc long, Sc_1_ ending beyond fork of Rs; Sc_2_ a greater distance before tip of Sc_1_, Sc_1_ 1.5–2 times as long as Sc_2_; basal section of CuA_1_ 1/2–2/3 of its own length before fork of M. Halter length 1.7–1.8 mm, white with knob dark brown.

***Abdomen*** (Fig. 1a). Tergites 1–4 brownish black with lateral regions brown, tergites 5–8 black. Sternites 1–3 brown, sternites 4–8 black.

**Figure 3.**
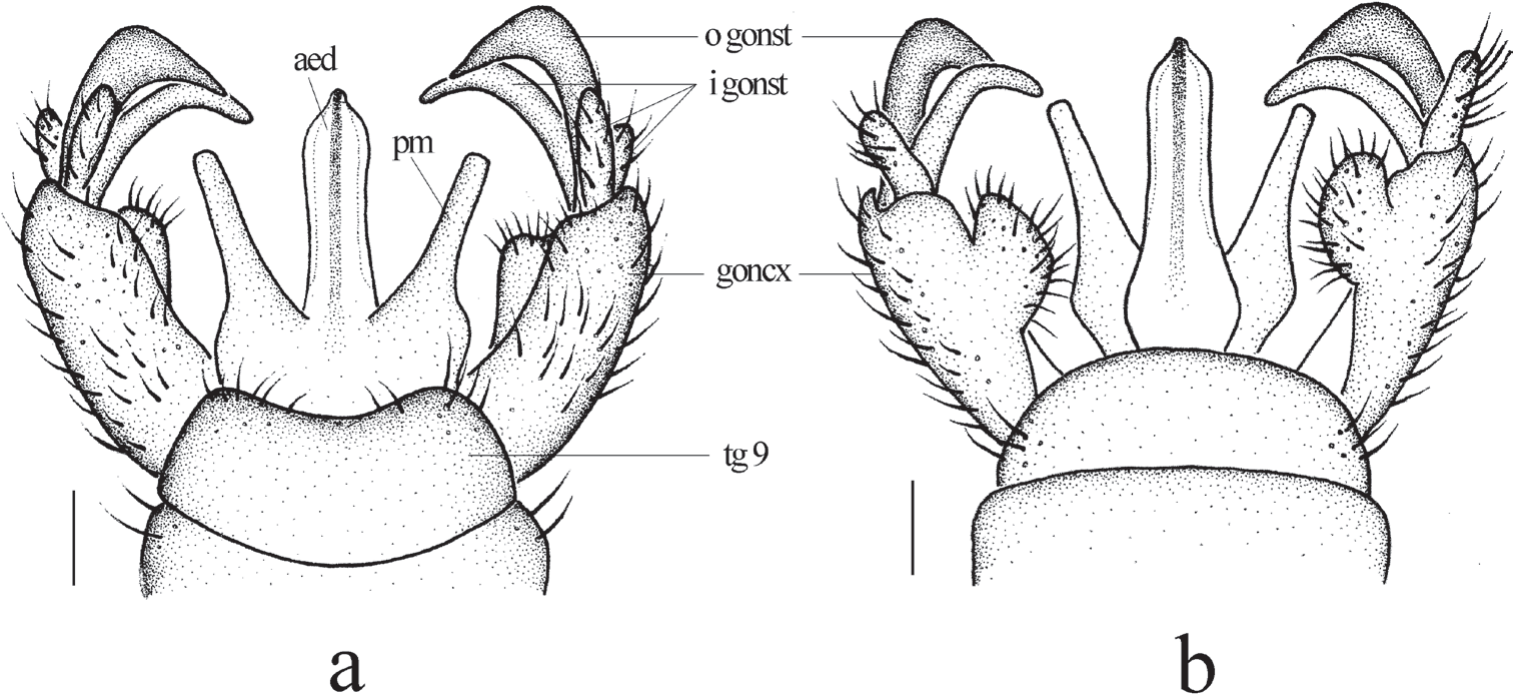
Metalimnobia (Metalimnobia) bicolor sp. nov. **a** male hypopygium, dorsal view **b** male hypopygium, ventral view. Scale bars: 0.2 mm.

***Hypopygium*** (Fig. 3). Posterior margin of ninth tergite emarginate, each lobe with several setae. Gonocoxite slender with a large, elongate and caudally curved ventro-mesal lobe. Outer gonostylus broad, arched at 2/3 length, outer third narrowing into a flattened spine. Inner gonostylus divided into three lobes: innermost lobe long, slender and curved; dorsal fleshy lobe fingerlike with long setae; ventral fleshy lobe oval with long setae. Paramere wide basally with distal part long and nearly straight, tip flattened and bare. Aedeagus long, slightly enlarged before tip.

**Female. *Body*** length 12.0 mm, wing length 11.0 mm. Similar to male, but tergites 6–9 brownish black with lateral regions brown, sternites 5–9 brown. Tenth tergite (Fig. 1d) brown. Cercus dark brown, base wide, tip pointed. Hypogynial valve dark brown with middle area of posterior half paler, tip reaching middle of cercus.

##### Distribution.

China (Sichuan).

##### Etymology.

The specific name refers to the wing spots at the origin and fork of Rs which have brown upper parts and brownish black lower parts.

##### Remarks.

**Figure 4.**
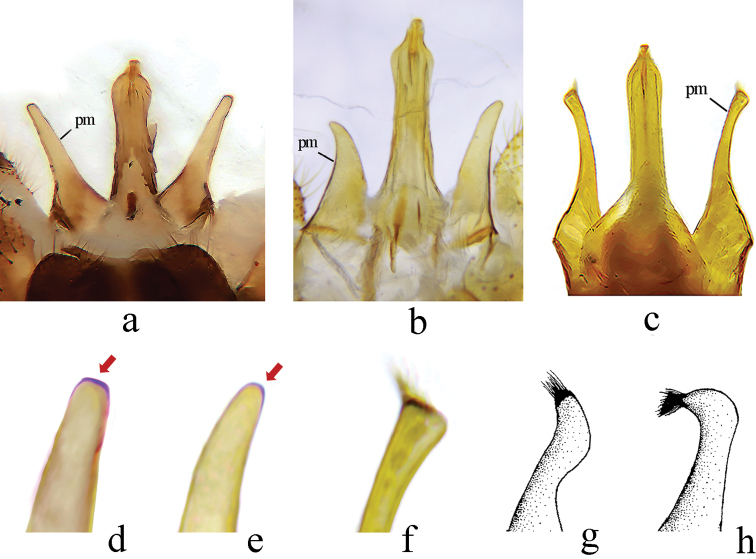
Details of male genitalia of *Metalimnobia***a–c** parameres and aedeagus, dorsal view **a**M. (M.) bicolor sp. nov. **b**M. (M.) tenua**c**M. (M.) caudifusca sp. nov. **d–h** tip of paramere **d**M. (M.) bicolor sp. nov. **e**M. (M.) tenua**f**M. (M.) caudifusca sp. nov. **g**M. (M.) quadrimaculata**h**M. (M.) rectangularis**g, h** after Mao and Yang 2010.

Metalimnobia (M.) bicolor sp. nov. can readily be distinguished from all other *Metalimnobia* crane flies known from China by the wing spots at the origin and fork of Rs. This species has a somewhat similar wing to the widespread M. (M.) tenua but can be easily distinguished from the latter by the antenna with dark brown scape and brownish black pedicel, the mostly brownish black pleuron, the brown coxae, and the paramere being wide basally with a long and nearly straight distal part and a flattened tip (Figs 3, 4a, 4d). In M. (M.) tenua, the scape and pedicel of the antenna are yellowish brown, the pleuron is mostly yellowish brown, the coxae are pale yellow, and the paramere is subtriangular with an acute tip (Fig. 4b, e).

#### Metalimnobia (Metalimnobia) bifasciata

Taxon classificationAnimaliaDipteraLimoniidae

(Schrank, 1781)

999365B6-82C2-574C-91C6-A9AEC61FDCBA

Tipula bifasciata Schrank, 1781: 429. Type locality: Austria, Linz
Limonia xanthoptera Meigen, 1804: 56. Type locality: not given
Metalimnobia vittata Matsumura, 1911: 63. Type locality: Russia, Sakhalin, Naiptchi
Limnobia avis avis Alexander, 1918: 444. Type locality: Japan, Honshu, Shinano, Takuhara
Limnobia avis flavoabdominalis Alexander, 1918: 445. Type locality: Japan, Honshu

##### Specimens examined.

1 male, China: Sichuan, Yanyuan, Lugu Lake (27°43'00"N, 100°54'18"E, 2673 m), 2019.VII.19, Liang Wang (light trap).

##### Diagnosis.

Pleuron yellow. Femora yellow with apical brown rings. Wing yellowish without spot at base. Sc_1_ ending close to fork of Rs, Sc_2_ longer than Sc_1_; basal section of CuA_1_ close to or beyond fork of M. Outer gonostylus broad, arched at 2/3 length, outer angle extended and darkened. Inner gonostylus undivided. Paramere with distal part long and nearly straight, tip blunt with several hairs on inside.

##### Distribution

**(new record in bold).** China (Beijing, Guizhou, Hebei, Heilongjiang, Hubei, Jilin, Liaoning, Ningxia, Shaanxi, Shanxi, **Sichuan**); Austria, Belarus, Belgium, Bulgaria, Croatia, Czech Rep., Denmark, Estonia, Finland, France, Germany, Georgia, Great Britain, Hungary, Ireland, Italy, Japan, Kazakhstan, ?Latvia, Lithuania, Luxembourg, Mongolia, Netherlands, North Korea, Norway, Poland, Romania, Russia, Serbia, Slovakia, Slovenia, South Korea, Sweden, Switzerland, Tajikistan, Turkey, Ukraine (Oosterbroek 2020).

##### Remarks.

Metalimnobia (M.) bifasciata is widely spread in the Palearctic and Oriental regions. In China, this species is known in many provinces and is now recorded in Sichuan for the first time. For descriptions and illustrations of this species, see Alexander (1918), Boardman (2007), Mao and Yang (2010), Matsumura (1911), Nakamura (2006), Podenas et al. (2006), Podenas and Gelhaus (2007) and Podenas and Byun (2016).

#### Metalimnobia (Metalimnobia) caudifusca sp. nov.

Taxon classificationAnimaliaDipteraLimoniidae

0F2A529A-FAE8-560B-A372-CBE501B0E5DC

http://zoobank.org/FD4F8AD7-031D-48A5-8A7D-7D983C27F7EB

Figures 5 , 6

##### Type material.

***Holotype*** male, China: Xizang, Bayi, Tibet agriculture and Animal Husbandry University (29°39'46"N, 94°20'43"E, 3000 m), 2014.VIII.22–IX.18, Baohai Wang (Malaise trap). ***Paratypes***: 1 male, same data as holotype. 1 male, China: Xizang, Bayi, Lulang (29°43'10"N, 94°42'06"E, 3800 m), 2009.VIII.2, Maoling Sheng.

##### Diagnosis.

Pleuron yellow. Fore femur yellow with apical brown ring; mid and hind femora each with two rings, apical ring brown, subapical ring pale brown and spreading over half of femur. Wing grayish without spot at base. Sc_1_ ending beyond fork of Rs, Sc_2_ shorter than Sc_1_; basal section of CuA_1_ before fork of M. Inner gonostylus divided into three lobes; dorsal fleshy lobe arched at 2/3 length, tip pointed. Paramere with distal part long, slender and slightly curved outwards, tip angulate with tuft of hairs.

##### Description.

**Male. *Body*** length 9.5–10.5 mm, wing length 11.5–13.0 mm.

**Figure 5.**
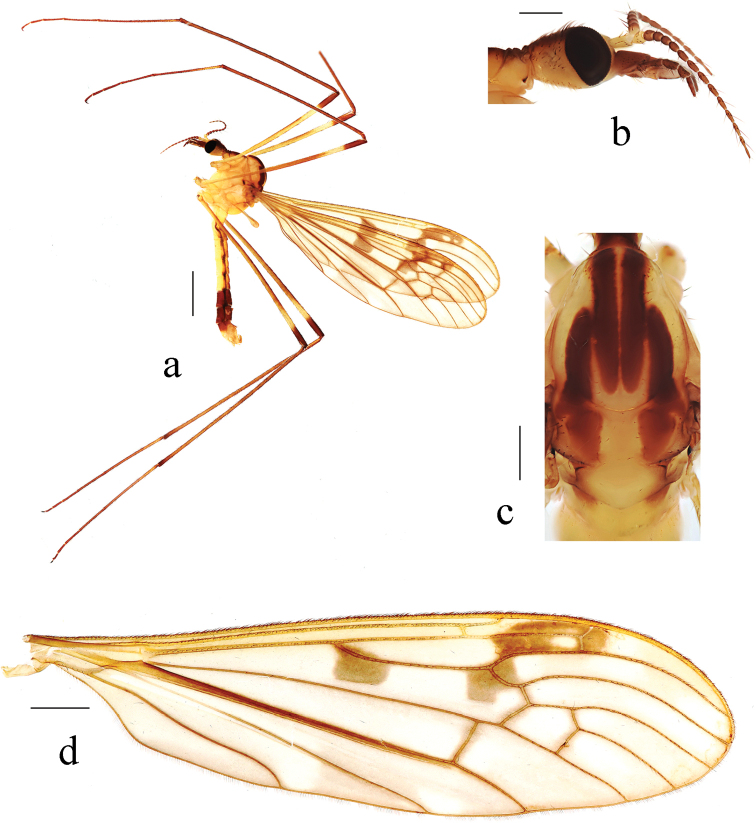
Metalimnobia (Metalimnobia) caudifusca sp. nov. **a** male habitus, lateral view **b** head, lateral view **c** thorax, dorsal view **d** wing. Scale bars: 2.0 mm (**a**); 0.5 mm (**b, c**); 1.0 mm (**d**).

***Head*** (Fig. 5b) brownish yellow with vertex brown and frons yellow. Setae on head brown. Antenna length 2.2–2.5 mm, yellow with flagellomeres brown. Scape cylindrical; pedicel nearly globose; flagellomeres oval with long setae, each flagellomere slightly narrower than previous one, terminal flagellomere elongated. Rostrum brown with dark brown setae. Palpus brown with dark brown setae.

***Thorax*** (Fig. 5c). Pronotum brown with lateral regions brownish yellow. Prescutum yellow with four broad, brown longitudinal stripes, lateral stripes about 1/2 length of median stripes. Scutum yellow, each lobe with two large pale brown spots, anterior spot connected with lateral stripe of prescutum. Scutellum yellow with lateral regions pale brown. Mediotergite yellow, base with two pale brown spots. Pleuron (Fig. 5a) yellow. Setae on thorax dark brown. Coxae yellow. Trochanters pale yellow. Fore femur yellow, tip with brown ring; mid and hind femora each yellow with two rings, apical ring brown, subapical ring pale brown and spreading over half of femur. Tibiae brown. Tarsi brown, bases of first tarsal segments slightly paler. Setae on legs dark brown. Wing (Fig. 5d) grayish with pale brownish yellow to pale brown pattern: large, pale brown spots at origin and fork of Rs; stigma pale brown with middle area paler; very pale brown seams along cord, m-m, basal section of M_3_ and base of CuA; obscure, irregular pale brownish yellow clouds in most cells. Veins pale brownish yellow, darker in clouded areas. Venation: Sc long, Sc_1_ ending beyond fork of Rs; Sc_2_ a greater distance before tip of Sc_1_, Sc_1_ 1.2–3 times as long as Sc_2_; basal section of CuA_1_ 1/4–1/2 of its own length before fork of M. Halter length 1.7–1.9 mm, pale yellow.

***Abdomen*** (Fig. 5a). Tergites 1–5 yellow to brownish yellow, tergites 6–8 brown. Sternites 1–6 yellow, sternites 6–8 brown.

**Figure 6.**
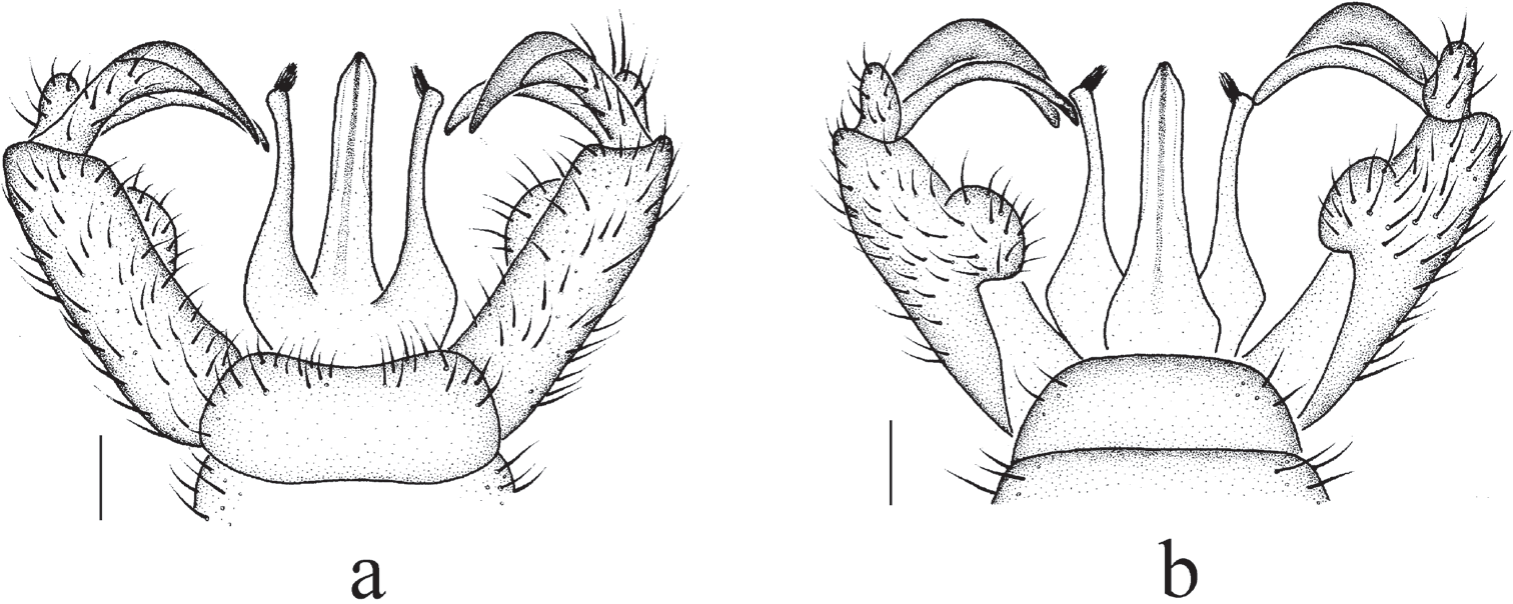
Metalimnobia (Metalimnobia) caudifusca sp. nov. **a** male hypopygium, dorsal view **b** male hypopygium, ventral view. Scale bars: 0.2 mm.

***Hypopygium*** (Fig. 6). Posterior margin of ninth tergite emarginate, each lobe with several setae. Gonocoxite slender with short, rounded and apically blunt ventro-mesal lobe. Outer gonostylus broad, arched at 2/3 length, outer third narrowing into a flattened spine. Inner gonostylus divided into three lobes: innermost lobe long, slender and curved; dorsal fleshy lobe long and stout with long setae, arched at 2/3 length, tip pointed; ventral fleshy lobe oval with long setae. Paramere wide basally with distal part long, slender and slightly curved outwards, tip angulate with tuft of hairs. Aedeagus long and slender.

**Female.** Unknown.

##### Distribution.

China (Xizang/Tibet).

##### Etymology.

The specific name refers to the caudal segments of the abdomen being uniformly brown.

##### Remarks.

Some *Metalimnobia* crane flies that occur in China also have a tuft of hairs at the tip of the parameres (e.g., *bifasciata*, *quadrimaculata*, *rectangularis* and *xanthopteroides*). However, the wing of M. (M.) caudifusca sp. nov. is grayish, while the wings of both M. (M.) bifasciata and M. (M.) xanthopteroides xanthopteroides are yellowish. Metalimnobia (M.) caudifusca sp. nov. can also be easily distinguished from M. (M.) quadrimaculata and M. (M.) rectangularis by the wing pattern (*caudifusca* has no spot at the base of the wing, but *quadrimaculata* and *rectangularis* have a spot at the base of their wings) and the shape of the paramere (Fig. 4c, f–h). Notably, the type specimens of M. (M.) yunnanica deposited in USNM and NHM were examined and showed that its wing had pale spots at the base.

#### Metalimnobia (Metalimnobia) quadrinotata

Taxon classificationAnimaliaDipteraLimoniidae

(Meigen, 1818)

DEA3A8C8-F464-5335-8391-9573B6B56A49

Limnobia quadrinotata Meigen, 1818: 144. Type locality: not given (?near Stolberg [Germany])
Limnobia variegata Macquart, 1826: 153. Type locality: northern France

##### Specimens examined.

1 male, China: Jilin, Antu, Mount Changbai, Lvyuantan (42°03'43"N, 128°04'05"E, 1775 m), 2015.VIII.6, Zehui Kang. 1 male 1 female, China: Hebei, Xinglong, Mount Wuling, main peak (40°35'30"N, 117°29'29"E, 1780 m), 2017.VIII.9, Liang Wang. 2 males, China: Neimenggu, Genhe, Hanma National Nature Reserve, Central Management Station (51°37'29"N, 122°26'34"E, 1200 m), 2014.VIII.1, Li Shi.

##### Diagnosis.

Pleuron brownish yellow. Femora each brownish yellow with two rings, apical ring black, subapical ring paler and spreading over half of femora. Wing brownish with one or more spots at base. Sc_1_ ending beyond fork of Rs, Sc_2_ longer than Sc_1_; basal section of CuA_1_ before fork of M. Inner gonostylus divided into three lobes. Paramere with distal part long and curved ventrally, tip flattened and bare.

##### Distribution

**(new records in bold).** China (Gansu, **Hebei**, Heilongjiang, **Jilin**, **Neimenggu**, Xinjiang); Armenia, Austria, Belarus, Belgium, Czech Rep., Denmark, Estonia, Finland, France, Georgia, Germany, Great Britain, Hungary, Ireland, Italy, Kazakhstan, Kyrgyzstan, Latvia, Lithuania, Luxembourg, Macedonia, Mongolia, Montenegro, Netherlands, North Korea, Norway, Poland, Romania, Russia, Slovakia, Slovenia, South Korea, Spain, Sweden, Switzerland, Ukraine (Oosterbroek 2020).

##### Remarks.

Metalimnobia (M.) quadrinotata is widespread in the Palearctic region. In China, this species was previously known in three provinces and is now recorded in Hebei, Jilin and Neimenggu for the first time. For descriptions and illustrations of this species, see Savchenko (1985), Podenas et al. (2006), Podenas and Gelhaus (2007), Mao and Yang (2010) and Podenas and Byun (2016).

#### Metalimnobia (Metalimnobia) tenua

Taxon classificationAnimaliaDipteraLimoniidae

Savchenko, 1976

CF0A61B5-06D0-5378-AFEE-10F3374C9F02

Metalimnobia quadrinotata tenua Savchenko & Krivolutskaya, 1976: 151. Type locality: Russia, several localities in Eastern Siberia and the Far East

##### Specimens examined.

1 male, China: Sichuan, Pingwu, Wanglang National Nature Reserve, Shuizhagou (32°54'16"N, 104°09'34"E, 2447 m), 2016.VII.20, Yizhe Li.

##### Diagnosis.

Pleuron brownish yellow, variegated by darker areas. Femora each brownish yellow with two rings, apical ring black, subapical ring slightly paler and spreading over half of femora. Wing brownish with two relatively large spots at base. Sc_1_ ending beyond fork of Rs, Sc_2_ shorter than Sc_1_; basal section of CuA_1_ before fork of M. Inner gonostylus divided into three lobes. Paramere subtriangular, tip slightly acute and bare (Fig. 4b, e).

##### Distribution

**(new record in bold).** China (Ningxia, **Sichuan**); Austria, Czech Rep., Italy, Finland, Japan, Kazakhstan, Mongolia, Norway, Russia, Slovakia, Sweden (Oosterbroek 2020).

##### Remarks.

Metalimnobia (M.) tenua is widespread in the Palearctic region. In China, this species was previously only known in Ningxia and is now recorded in Sichuan for the first time. For descriptions and illustrations of this species, see Savchenko and Krivolutskaya (1976), Savchenko (1983), Podenas and Gelhaus (2007) and Mao and Yang (2010).

## Supplementary Material

XML Treatment for Metalimnobia (Metalimnobia) bicolor

XML Treatment for Metalimnobia (Metalimnobia) bifasciata

XML Treatment for Metalimnobia (Metalimnobia) caudifusca

XML Treatment for Metalimnobia (Metalimnobia) quadrinotata

XML Treatment for Metalimnobia (Metalimnobia) tenua

## References

[B1] Alexander CP (1918) Records of Japanese crane-flies (Diptera). Annals of the Entomological Society of America 11: 443–449. 10.1093/aesa/11.4.443

[B2] Alexander CP (1933) New or little-known Tipulidae from eastern Asia (Diptera). XIV. Philippine Journal of Science 51: 507–544.

[B3] Alexander CP (1965) New or little-known Tipulidae from Madagascar (Diptera). Transactions of the American Entomological Society 91: 39–83.

[B4] Alexander CP (1976) New or insufficiently known African crane flies. V. (Diptera: Tipulidae). Studia Entomologica (N.S.) 19: 315–362.

[B5] Alexander CP Byers GW (1981) Tipulidae. In: McAlpine JF Peterson BV Shewell GE Teskey HJ Vockeroth JR Wood DM (Eds) Manual of Nearctic Diptera (Vol.I). Biosystematic Research Centre, Ottawa, 153–190.

[B6] Boardman P (2007) A Provisional Account and Atlas of the Craneflies of Shropshire. Privately published, Oswestry, 96 pp.

[B7] Dienske JW (1987) An illustrated key to the genera and subgenera of the western palaearctic Limoniidae (Insecta, Diptera) including a description of the external morphology. Stuttgarter Beitrage zur Naturkunde (A) 409: 1–52.

[B8] Edwards FW (1928) Some nematocerous Diptera from Yunnan and Tibet. Annals and Magazine of Natural History (10)1: 681–703. 10.1080/00222932808672840

[B9] Linnaeus C (1760) Fauna Svecica sistens animalia Sveciae regni: Mammalia, Aves, Amphibia, Pisces, Insecta, Vermes. Distributa per classes & ordines, genera & species, cum differentiis specierum, synonymis auctorum, nominibus incolarum, locis natalium, descriptionibus insectorum. Editio altera, auctior. Salvii, Stockholmiae [= Stockholm], 578 pp 10.5962/bhl.title.46380

[B10] Macquart PJM (1826) Insectes Diptères du nord de la France. [Tome I.] Tipulaires. Mémoires de la Société Royale des Sciences, de l’Agriculture et des Arts, de Lille 1823–1824, 59–224. 10.5962/bhl.title.8146

[B11] Mao M Yang D (2010) Species of the genus *Metalimnobia* Matsumura from China (Diptera, Limoniidae). Zootaxa 2344: 1–16. 10.11646/zootaxa.2344.1.1

[B12] Matsumura S (1911) Erster Beitrag zur Insekten-Fauna von Sachalin. Journal of the College of Agriculture, Tohoku Imperial University, Sapporo 4: 1–415.

[B13] McAlpine JF (1981) Morphology and terminology, Adults. In: McAlpine JF Peterson BV Shewell GE Teskey HJ Vockeroth JR Wood DM (Eds) Manual of Nearctic Diptera (Vol.I). Biosystematic Research Centre, Ottawa, 9–63.

[B14] Meigen JW (1804) Klassifikazion und Beschreibung der europäischen zweiflügeligen Insekten (Diptera Linn.). Erster Band. Abt. I & II. Reichard, Braunschweig [= Brunswick], [i–xxviii], 1–152; 2, [i–vi], 153–314.

[B15] Meigen JW (1818) Systematische Beschreibung der bekannten europäischen zweiflügeligen Insekten. F.W. Forstmann, Aachen, 1, [i–xxxvi,] 324 pp. 10.5962/bhl.title.12464

[B16] Nakamura T (2006) Diptera of the Nasu Imperial Villa, Tochigi, Japan. Flora and Fauna of the Nasu Imperial Villa. Tochigi Prefectural Museum, Utsunomiya, 167–170.

[B17] Oosterbroek P (2020) Catalogue of the Craneflies of the World (Diptera, Tipuloidea, Pediciidae, Limoniidae, Cylindrotomidae, Tipulidae). http://ccw.naturalis.nl/ [Accessed on 2020-11-11]

[B18] Podenas S Byun HW (2016) *Metalimnobia* crane flies (Diptera: Limoniidae) from Korea. Zootaxa 4132: 330–346. 10.11646/zootaxa.4132.3.2 27395675

[B19] Podenas S Geiger W Haenni JP Gonseth Y (2006) Limoniidae & Pediciidae de Suisse. Fauna Helvetica 14: 1–375.

[B20] Podenas S Gelhaus JK (2007) Identification keys for Limoniinae (Diptera, Limoniidae) of Mongolia and adjacent territories. Vilnius, Lithuania, 85 pp.

[B21] Riedel MP (1917) H. Sauters Formosa-Ausbeute. Nematocera polyneura (Dipt.). 3. Archiv fur Naturgeschichte 82(A)(5): 109–116.

[B22] Salmela J Starý J (2009) Description of Metalimnobia (Metalimnobia) charlesi sp. n. from Europe (Diptera, Limoniidae). Entomologica Fennica 19: 268–272. 10.33338/ef.84444

[B23] Savchenko EN (1983) Limoniidae of South Primorye. Akademiy Nauk Ukrainskoy SSR, I.I. Schmalhausen Institute of Zoology of Academy of Sciences of Ukraine. Naukova Dumka, Kiev, 156 pp.

[B24] Savchenko EN (1985) Komary-limoniidy [limoniid-flies]. Subfamily Limoniinae. Fauna Ukrainy 14(4): 1–180.

[B25] Savchenko EN Krivolutskaya GO (1976) Limoniidae of the south Kuril Islands and south Sakhalin. Akademiy Nauk Ukrainskoy SSR, Kiev, 160 pp.

[B26] Schrank FP (1781) Enumeratio insectorum Austriae indigenorum. V.E. Klett & Franck, Avguvstiae Vindelicorum [Augsburg], [i–xxiv,] 548 pp.

